# Conversion of Carbon Dioxide into Chemical Vapor Deposited Graphene with Controllable Number of Layers via Hydrogen Plasma Pre-Treatment

**DOI:** 10.3390/membranes12080796

**Published:** 2022-08-18

**Authors:** Yotsarayuth Seekaew, Nantikan Tammanoon, Adisorn Tuantranont, Tanom Lomas, Anurat Wisitsoraat, Chatchawal Wongchoosuk

**Affiliations:** 1 Graphene and Printed Electronics Research Division (GPERD), National Security and Dual-Use Technology Center, National Science and Technology Development Agency, 111 Thailand Science Park, Phahon Yothin Road, Klong Nueng, Klong Luang, Phathum Thani 12120, Thailand; 2 Department of Physics, Faculty of Science, Ramkhamhaeng University, Bang Kapi, Bangkok 10240, Thailand; 3 Department of Physics, Faculty of Science, Kasetsart University, Chatuchak, Bangkok 10900, Thailand

**Keywords:** graphene, carbon dioxide, hydrogen plasma, chemical vapor deposition

## Abstract

In this work, we report the conversion of carbon dioxide (CO_2_) gas into graphene on copper foil by using a thermal chemical vapor deposition (CVD) method assisted by hydrogen (H_2_) plasma pre-treatment. The synthesized graphene has been characterized by Raman spectroscopy, X-ray diffraction, scanning electron microscopy, and transmission electron microscopy. The results show the controllable number of layers (two to six layers) of high-quality graphene by adjusting H_2_ plasma pre-treatment powers (100–400 W). The number of layers is reduced with increasing H_2_ plasma pre-treatment powers due to the direct modification of metal catalyst surfaces. Bilayer graphene can be well grown with H_2_ plasma pre-treatment powers of 400 W while few-layer graphene has been successfully formed under H_2_ plasma pre-treatment powers ranging from 100 to 300 W. The formation mechanism is highlighted.

## 1. Introduction

Carbon dioxide (CO_2_) is an important greenhouse gas in the atmosphere [1]. It helps to trap heat on our Earth’s surface and supports the growth of plants in the agricultural cycle [2,3]. However, high emission of CO_2_ produced by human activities, such as the combustion of fossil fuels to produce electricity, has recently caused serious problems in the form of global warming and climate change [4]. Moreover, exposure to atmospheric CO_2_ at high levels produces a variety of human health effects [5]. To mitigate the adverse effects of high CO_2_ emissions, there has been increasing research interest in CO_2_ reduction and utilization [6,7,8]. One of the most compelling utilizations of CO_2_ is to convert it into valuable products such as carbon-based nanomaterials, such as carbon quantum dots, inorganic nanoparticles, carbon nanotubes (CNTs) and graphene [9,10,11,12,13,14]. For examples, Kim et al. [15] synthesized the multi-walled CNTs by chemical vapor deposition (CVD) with NaBH_4_ reductant and NiCl_2_ catalyst. Ren et al. [16] demonstrated the preparation of carbon nanofibers via molten carbonate electrolysis from atmospheric CO_2_. Molina-Jirón et al. [17] reported the growth of graphene via atmospheric-pressure CVD with a catalytic Cu-Pd alloy. Licht et al. [18] produced carbon nanofibers and CNTs from CO_2_ using a solar thermal electrochemical process. Wang et al. [19] presented a transformation of CO_2_ into a carbon nano-scaffold by using electrolysis in molten carbonate. Until now, the development of new methods or technologies for the conversion of CO_2_ to valuable nanomaterials has been in great demand since CO_2_ emission has become one of the biggest global concerns, and as net zero by 2050 is the goal.

Graphene is one of the well-known two-dimensional (2D) materials that has exceptional physical, chemical, and electrical properties [20,21,22,23]. Currently, graphene has been widely applied in several applications including nanoelectronics, flexible electronics, batteries, super-capacitors, solar cells, gas sensors, membranes, and chemical sensors [11,24,25,26,27,28,29,30]. Graphene can be grown by numerous methods including CVD, mechanical exfoliation, chemical oxidation/reduction and electrolytic exfoliation [31,32,33]. Among them, CVD is one of the most popular graphene growth methods because it can produce a high-quality monolayer and few-layer graphene [34,35,36,37,38]. The basic principle of thermal CVD relies on the decomposition of gas molecules, such as methane [39], acetylene [40], ethylene [36], and ethanol [41], to react with some metal catalysts and induce graphene growth. Without metal catalysts on substrates, a high temperature of up to 1650 °C is required to overcome the large energy barrier for graphene nucleation [42].

In this work, we report the conversion of CO_2_ into graphene on copper foil substrates using a CVD method with hydrogen (H_2_) plasma pre-treatment. To our best knowledge, this is the first work to investigate the effects of H_2_ plasma pre-treatment to convert CO_2_ into graphene with the controllable few layers. Our finding demonstrates the important role of H_2_ plasma pre-treatment in the formation of few-layer graphene (two–six layers) by adjusting radio frequency (rf) powers for plasma pre-treatments (100–400 W).

## 2. Materials and Methods

**Figure 1:**
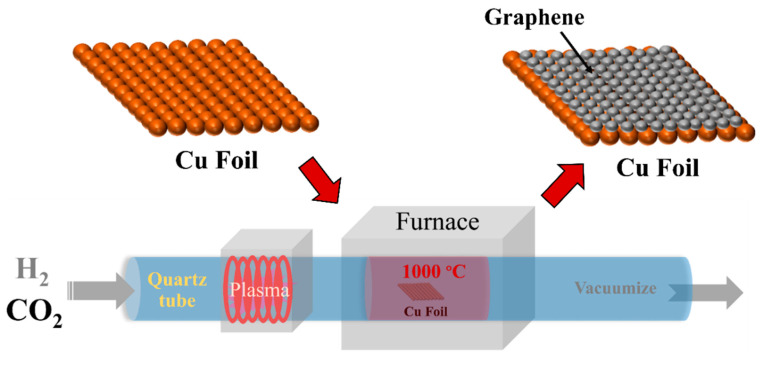
Schematic diagram for the conversion of CO_2_ to graphene on a Cu foil by the chemical vapor deposition (CVD) method.

Copper (Cu) foil (25 µm thick, 99.98% metals basis) was purchased from Sigma-Aldrich Co., LLC, Darmstadt, Germany. The graphene was grown on the Cu foil by CVD method using CO_2_ gas (99.999% purity) as a carbon source, as shown in Figure 1. The growth process was conducted using a customized thermal CVD system integrated with an inductively coupled plasma system (planarGROW-4S, planarTECH LLC, The Woodlands, TX, USA). The distance from the plasma coil to the sample was ~75 cm. Before the Cu foil was loaded into a 4″ horizontal quartz tube of the CVD system, it was washed in ethanol solution for 10 min under ultrasonication and dried in air at room temperature. After the loading of samples, 150 sccm of H_2_ flowed into the CVD quartz tube while the reactor was heated to 1000 °C at a pressure of 1 Torr. At 1000 °C, the Cu foil surface was treated using H_2_ plasma generated by rf power for 30 min. The rf power was adjusted from 100 to 400 W in order to investigate the effects of H_2_ plasma pre-treatment. Next, a mixture of CO_2_ (50 sccm) and H_2_ (200 sccm) was applied at a working pressure of 2 Torr for 30 min for graphene growth on Cu foils. After the graphene growth stage, the CVD quartz tube reactor was cooled down to room temperature under an H_2_ flow of 150 sccm at 1 Torr. In the CVD process, the heating rate was set at 15 °C/min while the cooling rate was set at 10 °C/min. The graphene samples were characterized by field-emission scanning electron microscopy (FE-SEM: SU8030, Hitachi, Tokyo, Japan), X-ray diffraction (XRD: D8 Advance, Bruker, MA, USA), transmission electron microscopy (TEM: JEM-2100 Plus, JEOL, Tokyo, Japan), and Raman spectroscopy (InVia Raman Microscope, Renishaw, West Dundee, IL, USA) using a laser with an excitation wavelength of 785 nm.

## 3. Results and Discussion

**Figure 2:**
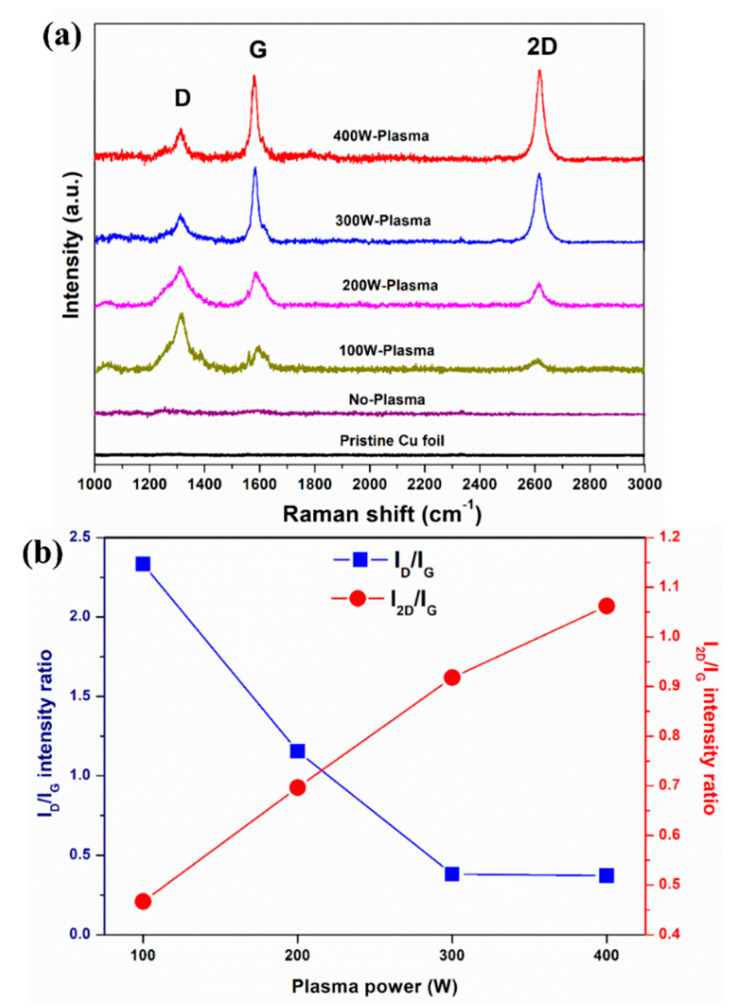
Raman spectra of (**a**) graphene growth on Cu foils pre-treated with different H_2_ rf plasma powers and (**b**) their intensity ratio values of I_D_/I_G_ and I_2D_/I_G_. It should be noted that the H_2_ gas still flowed over the sample with the rf power off in the case of “No-Plasma”.

After the growth process, all samples were initially characterized by Raman spectroscopy as presented in Figure 2. It is evident that only the samples with H_2_ plasma pre-treatment (rf power = 100–400 W) exhibit D, G and 2D peaks at around 1300, 1580 and 2600 cm^−1^, respectively (Figure 2a). It is well known that the D peak is associated with lattice defects of the graphene structure while the G peak corresponds to primary sp^2^-hybridized carbon bonds in graphene. The 2D peak is the second order of the D band relating to one boundary defect in graphene [43,44]. Therefore, they confirm the formation of graphene on Cu surfaces with H_2_ plasma pre-treatments. At the same growth condition, no graphene was observed on Cu surfaces without H_2_ plasma pre-treatment. This indicates that the H_2_ plasma pre-treatment plays an important role in modifying the Cu surface for graphene nucleation.

To investigate the quality and number of graphene layers, the intensity ratios of the 2D to G band (I_2D_/I_G_) and D to G band (I_D_/I_G_) were calculated from the Raman spectra and are displayed in Figure 2b. The I_2D_/I_G_ is known to be strongly related to the number of layers [34,45,46,47]. The I_2D_/I_G_ > 2 indicates the monolayer graphene while 1 < I_2D_/I_G_ < 2 and I_2D_/I_G_ < 1 refer to the bilayer and trilayer/few-layer graphene, respectively. In Figure 2b, the I_2D_/I_G_ increases from 0.47 to 1.06 with increasing rf plasma power from 100 W to 400 W, suggesting the formation of few-layer graphene and the decrease of the number of graphene layers to two on increasing the H_2_ plasma rf power to 400 W. Concerning the graphene quality, the I_D_/I_G_ decreases from 2.33 to 0.37 as the rf plasma power increases from 100 W to 400 W. The decrease of the I_D_/I_G_ intensity ratio implies the reduction of defect density. At the high plasma powers of 300–400 W, the I_D_/I_G_ intensity ratio is as low as ~0.37, indicating graphene structures with low defect levels [48].

**Figure 3:**
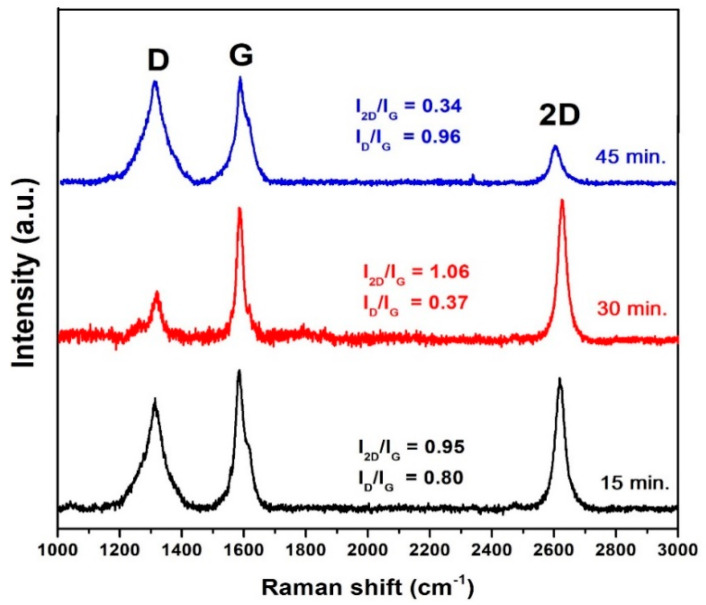
Raman spectra of graphene growth on Cu foils pre-treated with H_2_ rf plasma power of 400 W at different growth times.

The graphene growth evolution with respect to the growth times, including 15, 30 and 45 min, using the rf plasma power of 400 W, is shown in Figure 3. At all reaction times, the grown surfaces exhibit three main Raman peaks (D, G and 2D), indicating that graphene has already formed at 15 min, with I_2D_/I_G_ ~ 1 corresponding to the bilayer graphene structure. However, I_D_/I_G_ (0.80) at 15 min is relatively high compared with I_D_/I_G_ at 30 min (0.37) because the nucleated graphene is initially defective and these defects may be amended with additionally deposited atoms as the time progresses. For the extended growth time of 45 min, more layers of graphene are formed, leading to a significantly reduced I_2D_/I_G_ in accordance with the previous reports of other CVD graphene growth studies using different times [49,50,51].

**Figure 4:**
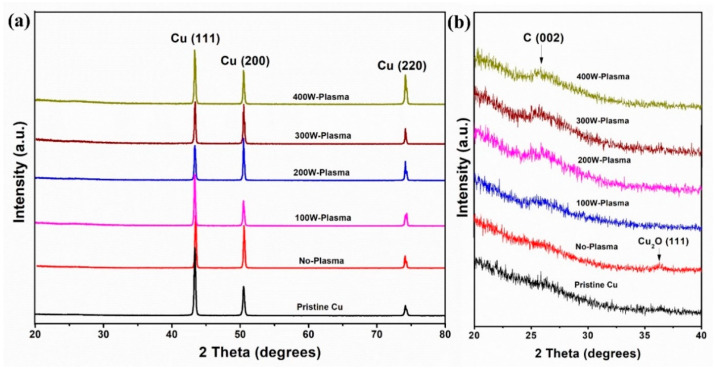
(**a**) XRD patterns and (**b**) zoom graph of XRD patterns of the pristine Cu and the graphene growth on Cu foils pre-treated with different H_2_ rf plasma powers.

To evaluate the effects of H_2_ rf plasma power during pre-treatment on graphene growth, the XRD patterns of the pristine Cu foil and the graphene growth on Cu foils pre-treated with different H_2_ rf plasma powers are displayed in Figure 4. As seen in Figure 4a, all samples exhibit three pronounced peaks located at 43.34°, 50.46° and 74.16°, corresponding to Cu (111), Cu (200) and Cu (220) (JCPDS No. 65-9026), respectively [52,53]. Interestingly, Cu_2_O (111) phase at 37° [54,55] arises in comparison with pristine Cu (unheated) and samples with H_2_ plasma pre-treatments after the sample was heated to the growth temperature (1000 °C) in the CVD system (Figure 4b). The formation of Cu_2_O during the CVD process can suppress the graphene’s growth. With H_2_ plasma pre-treatments, Cu_2_O is absent and the C (002) peak at ~26° is detected, dictating the formation of graphene on Cu foil without other defect peaks in accordance with the Raman results, which indicate high quality graphene structures.

**Figure 5:**
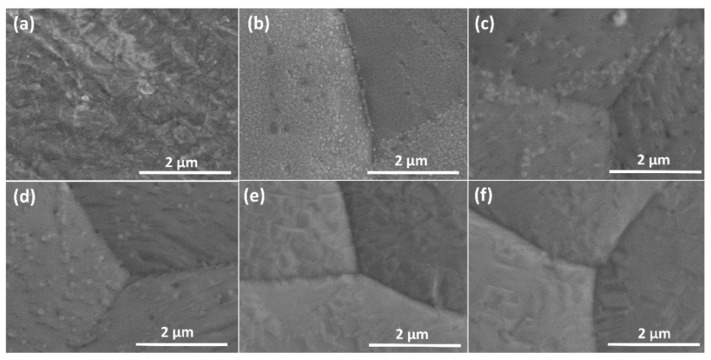
FE-SEM images of (**a**) pristine Cu foil, Cu foil after CVD growth with (**b**) no and with hydrogen plasma pre-treatment using rf powers of (**c**) 100 W, (**d**) 200 W, (**e**) 300 W and (**f**) 400 W for 30 min.

The detailed surface morphologies of graphene grown from CO_2_ on Cu foils pre-treated with varying H_2_ rf plasma powers are demonstrated in Figure 5. It clearly shows that the H_2_ plasma pre-treatment strongly affects the surface morphology of Cu foil. In this work, Cu foil acts as both catalyst and substrate. During the high-temperature CVD process, the recrystallization of Cu grain occurs and Cu_2_O is formed on the surface due to oxidation by residual oxygen. With H_2_ plasma pre-treatment, high rf powers can contribute to the removal of residual copper oxides on the surface as shown in Figure 5c–f. An increase of the rf plasma power results in a remarkable reduction in the density of residual oxides and enhances the carbon in-diffusion-controlled kinetics of CO_2_ flow in the reactor, leading to the formation of graphene. However, a large number of wrinkles are formed and some secondary nucleation always dominates. The wrinkles are caused by the discontinuous growth of monolayer graphene associated with the difference in thermal expansion between graphene and Cu [56,57]. Thus, only bilayer and few-layer graphene can be formed. In addition, the wrinkles of graphene on the Cu substrate are quite similar to the few-layer graphene wrinkles formed on other substrates such as Ni foam [58].

**Figure 6:**
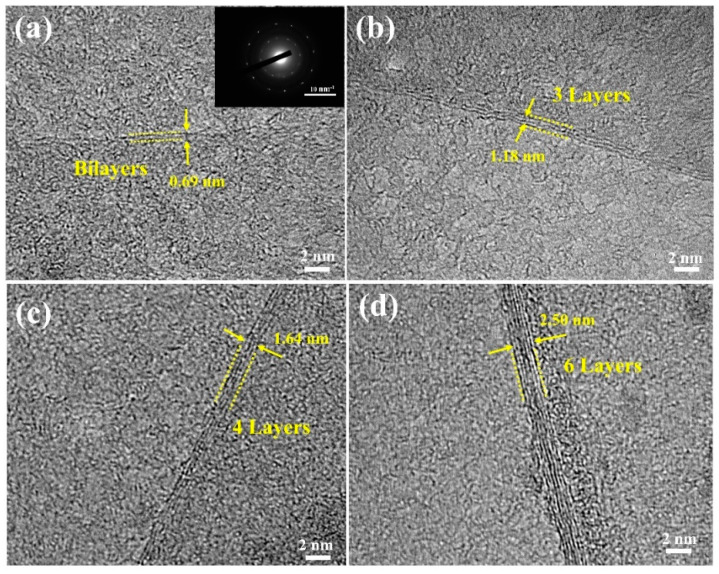
Representative high-resolution TEM images of graphene edges produced via hydrogen plasma pre-treatments with rf powers of (**a**) 400 W, (**b**) 300 W, (**c**) 200 W, and (**d**) 100 W. The inset shows a typical SAED pattern of the bilayer graphene.

The number of layers of graphene grown on Cu foils pre-treated with different plasma powers was verified by high-resolution (HR) TEM images as displayed in Figure 6. The HR-TEM images clearly show graphene fringes displaying bilayer, trilayers, four layers, and six layers in accordance with the Raman results of graphene grown on Cu foils pre-treated with the plasma powers of 400, 300, 200 and 100 W, respectively. The thickness of graphene with bilayer, trilayers, four layers, and six layers is estimated to be ~0.69 nm, 1.18 nm, 1.64 nm and 2.50 nm, respectively. In addition, the interlayer spacing of graphene sheets can be determined to be ~0.35 nm in agreement with many other publications [17,59,60]. The selected area electron diffraction (SAED) pattern, as illustrated in the inset of Figure 6a, presents two sets of six-fold reflection spots of a hexagonal lattice. This evidence confirms the bilayer graphene structure with high quality [61].

**Figure 7:**
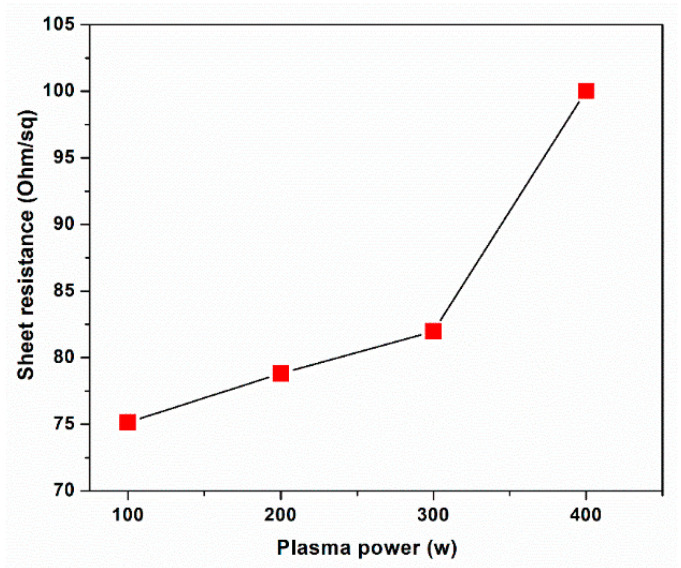
Sheet resistance of graphene on Cu foil as a function of the H_2_ plasma power.

**Figure 8:**
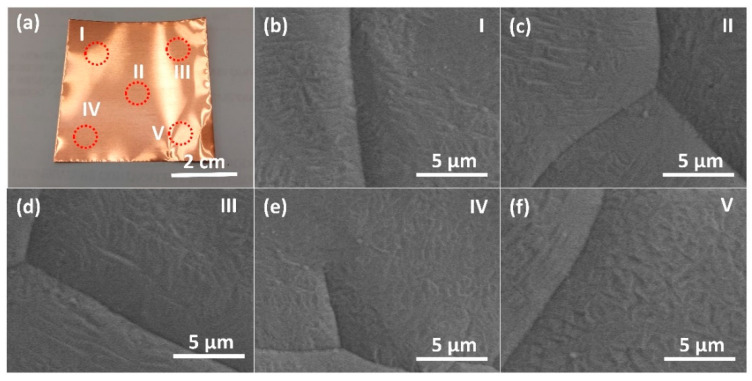
(**a**) Photograph of the real sample (Cu foil) after graphene growth with H_2_ plasma pre-treatment at 400 W for 30 min. (**b**–**f**) FE-SEM images of bilayer graphene at five different regions on the Cu foil.

The sheet resistances as a function of the rf plasma powers were also investigated as shown in Figure 7. The sheet resistance of graphene increases from ~75 to 100 Ω/sq on increasing the rf plasma power from 100 to 400 W. The number of graphene layers strongly correlates to band structure, energy gap, Fermi energy, and charge carriers, which directly affect the electrical conductivity [34,62,63,64]. From the characterization results, the number of graphene layers was six at 100 W and was reduced to two at 400 W. Both bilayer and multilayer graphene structures exhibit typical parabolic band structures associated with finite effective masses and charge carriers, which decrease with a decreasing number of graphene layers [34]. Bilayer graphene has more available electronic states in its valence band, leading to higher sheet resistances compared with multilayer graphene. In comparison with other substrates, the sheet resistance of graphene grown on Cu foil is smaller than that on PMMA and glass substrates (540–650 Ω/sq of bilayer and 300–350 Ω/sq of trilayer) [65,66]. The obtained low sheet resistance may be attributed to the uniformity of graphene film on the Cu surface and low defects compared with those produced by other methods [67]. To confirm the uniformity of graphene over the sample surface, FE-SEM images of bilayer graphene at five different regions on the Cu foil are displayed in Figure 8. It demonstrates that all regions show similar surface and wrinkle features of bilayer graphene on the Cu foil. Thus, the obtained bilayer graphene is highly uniform over the sample area.

The growth mechanism of CVD graphene depends on many factors, such as hydrocarbon gas source, pressure, flow rate, temperature, growth time, catalyst and substrate [68]. Several catalysts, such as Ni/Al_2_O_3_ [69], Cu-Pd [17], and NaCl–CaCl_2_–CaO [70], have been used to activate CO_2_ for the graphene growth. In this work, Cu foils act as both catalyst and substrate while only H_2_ plasma pre-treatment is used to activate the Cu catalyst with fixed temperature and time. Based on the characterization results, the H_2_ plasma power strongly affects the metal catalyst surface properties. Therefore, the formation of graphene with different numbers of layers is attributed to distinct metal catalyst surfaces pre-treated with different H_2_ plasma powers. In the synthesis step, CO_2_ is introduced as a carbon source for graphene growth. At high temperatures (~1000 °C), CO_2_ begins to decompose into carbon and oxygen atoms, generating CO and O [71], while CO_2_ can also directly react with H_2_ leading to the formation of H_2_O or other molecules (methane and methanol) [72,73]. The reaction for converting CO_2_ to graphene on the Cu surface can be described as CO_2_ + 2H_2_ → C + 2H_2_O [17]. However, copper oxide (Cu_2_O) formed on metal surfaces at high temperatures can suppress the diffusion of carbon species on the metal catalyst’s surface, preventing the nucleation of graphene. According to a previous study, graphene nucleation densities are low when Cu surfaces are relatively rough compared with the atomic thinness of the graphene [74]. From the results in this work, graphene cannot be formed without H_2_ plasma pre-treatment. The application of the H_2_ plasma pre-treatment on Cu foil can reduce residues on the surface and make the surface smoother. By increasing the H_2_ plasma power, residues are additionally removed, leading to an increasingly smooth Cu surface. The ingrained surface impurities act as nucleation sites for carbon adsorption during growth. Additional nucleation sites may be activated by H_2_ plasma pre-treatment to form the multilayer graphene. The H_2_ plasma power during pre-treatment can be thus used as a primary factor to control the number layers of graphene. In other words, C atoms dissociated from CO_2_ at a high growth temperature can diffuse the Cu surface into bulk to start the nucleation and growth of graphene on the catalyst’s surface. If the Cu surface confronts a contamination (oxidation of the unwanted impurities) before the precursor exposure, supersaturation of the surface is readily reached, limiting the nucleation of graphene. The H_2_ plasma pre-treatment can remove the contaminations on the catalyst surface and activate Cu active sites for graphene growth.

## 4. Conclusions

In conclusion, CO_2_ gas has successfully been converted into graphene films on Cu foils by way of a CVD method with H_2_ plasma pre-treatment. Raman spectroscopy, XRD, SEM, and TEM data demonstrate the formation of high-quality graphene with two–six layers on Cu foils. Without H_2_ plasma pre-treatment, a rough Cu surface with the cluster of oxide residues formed at a high growth temperature can suppress the diffusion of carbon species, resulting in no graphene nucleation. The introduction of the H_2_ plasma pre-treatment can remove residuals and enhance the CO_2_ flow kinetics on the Cu surface, assisting the nucleation and growth of graphene. The number of layers of graphene can be well controlled by varying the H_2_ plasma powers applied for the direct modification of metal catalyst surfaces before graphene growth. The proposed method requires no additional carrier gas and a catalyst for graphene growth from CO_2_. Therefore, it can be useful as an alternative way to convert CO_2_ greenhouse gas in the atmosphere into a valuable graphene film with a controllable number of layers.

## Data Availability

The data presented in this study are available on request from the corresponding author.
